# A Hybrid-Body Containing Constituents of Both P-Bodies and Stress Granules Forms in Response to Hypoosmotic Stress in *Saccharomyces cerevisiae*

**DOI:** 10.1371/journal.pone.0158776

**Published:** 2016-06-30

**Authors:** Khyati H. Shah, Sapna N. Varia, Laura A. Cook, Paul K. Herman

**Affiliations:** Department of Molecular Genetics, The Ohio State University, Columbus, Ohio 43210, United States of America; Texas A&M University, UNITED STATES

## Abstract

The cytoplasm of the eukaryotic cell is a highly compartmentalized space that contains a variety of ribonucleoprotein (RNP) granules in addition to its complement of membrane-bound organelles. These RNP granules contain specific sets of proteins and mRNAs and form in response to particular environmental and developmental stimuli. Two of the better-characterized of these RNP structures are the stress granule and Processing-body (P-body) that have been conserved from yeast to humans. In this report, we examined the cues regulating stress granule assembly and the relationship between stress granule and P-body foci. These two RNP structures are generally thought to be independent entities in eukaryotic cells. However, we found here that stress granule and P-body proteins were localized to a common or merged granule specifically in response to a hypoosmotic stress. Interestingly, these hybrid-bodies were found to be transient structures that were resolved with time into separate P-body and stress granule foci. In all, these data suggest that the identity of an RNP granule is not absolute and that it can vary depending upon the nature of the induction conditions. Since the activities of a granule are likely influenced by its protein constituency, these observations are consistent with the possibility of RNP granules having distinct functions in different cellular contexts.

## Introduction

The distinguishing feature of the eukaryotic cytoplasm is its subdivision into a number of functionally-distinct, membrane-enclosed organelles. However, it is becoming increasingly apparent that additional levels of compartmentalization exist within this aqueous space [1]. For example, specific ribonucleoprotein (RNP) granules have been shown to form in response to different environmental and developmental stimuli [2, 3]. These structures lack a limiting membrane but contain distinct sets of proteins and mRNAs at concentrations higher than the surrounding cytoplasm. These granules have been found to behave like liquid droplets in the cytoplasm and are thought to form as a result of multivalent interactions occurring between core proteins and mRNAs [1, 4, 5] Two of the best characterized of these structures are the evolutionarily-conserved Processing-body (P-body) and stress granule [6, 7]. Stress granules contain ribosomal subunits and translation factors and are thought to be a place of storage for mRNAs that will be translated following the removal of the ongoing stress [8–10]. In contrast, the conserved functions of P-bodies are less clear. Although these granules contain proteins involved in mRNA decay, several studies have found that mRNA turnover occurs normally in cells lacking P-body foci [11–13]. Other work has suggested that these foci could represent sites for mRNA storage, miRNA silencing and the regulation of virus production [14–16]. These suggestions typically arose as a result of experiments identifying novel granule constituents, like the Argonaute proteins [17]. These observations are thus consistent with the overarching notion that the functions of RNP granules are determined, at least in part, by the proteins resident within. Therefore, it is important that we define these constituents and the underlying mechanisms governing granule assembly.

Stress granules are dynamic structures that form in response to a variety of environmental and developmental stimuli [6, 18, 19]. These conditions are typically associated with a decrease in the rate of protein synthesis. In mammalian cells, granule assembly is triggered by the translation arrest that occurs following the stress-mediated phosphorylation of initiation factor eIF2α [20]. This block in initiation leads to polysome disassembly and the accumulation of 48S pre-initiation complexes that contain the naked mRNA [20]. This mRNA is subsequently bound by specific granule proteins that contain low-complexity, or intrinsically disordered domains. These domains are thought to mediate the aggregation of the pre-initiation complexes that ultimately results in the formation of the stress granule [21–24]. Although these granules have been conserved through evolution, the mechanisms that underlie their formation exhibit species- and stress-specific differences. For example, granule assembly in response to either heat stress or glucose deprivation appears to occur in an eIF2α-independent manner in both budding and fission yeast [8, 25–27]. In *S*. *pombe*, the cAMP-dependent protein kinase (PKA) is required for granule formation in response to a heat stress but not a hyperosmotic stress [27]. In contrast, PKA signaling is not required for stress granule formation in *S*. *cerevisiae* under any condition tested thus far [18]. No role for this signaling pathway has yet been uncovered in mammalian cells either. Finally, these RNP structures have also been found to contain a number of additional proteins including several involved in signal transduction [28–31]. These observations have led to the suggestion that these granules may have a role in the rewiring of signaling networks during periods of stress.

Although the P-body and stress granule are generally thought to be separate entities, previous work has described specific functional and physical interactions occurring between these structures. For example, studies with both yeast and mammalian cells suggest that specific mRNAs may traffic between P-bodies and stress granules [7, 32]. Consistent with these observations, these structures are induced by partially overlapping sets of environmental stimuli [25, 33–39]. However, it should be pointed out that the physiological relevance of granule induction is not clear for all of these conditions. With respect to P-bodies, previous work has suggested that the presence of these RNP granules is important for the long-term survival of nondividing or stationary phase yeast cells [40]. Working out the details of the relationships that exist between these and other RNP granules in the cytoplasm is likely to provide insight into the biological roles of these structures in the eukaryotic cell.

In this study, we examined the cues regulating stress granule assembly and the relationship between stress granule and P-body foci. Interestingly, the data indicate that the identity of an RNP granule can vary depending upon the nature of the inducing stress. In particular, we find that P-body and stress granule proteins localize to the same cytoplasmic granule in response to a hypoosmotic stress. The significance of this novel hybrid-body is discussed with respect to both conflicting localization studies in the literature and the biological consequences of forming such an alternative structure.

## Materials and Methods

### Yeast strains and growth conditions

Standard *Escherichia coli* and yeast growth conditions and media were used throughout this work. The yeast rich growth medium, YPAD, and the minimal YM and SC media have been described previously [41–43]. For the microscopy analysis, yeast cultures were grown at 30° in the appropriate medium with agitation for the indicated number of days. The carbon starvation experiments were performed in YP- or SC-based media lacking glucose as indicated. For the colocalization experiments, cells were typically transformed with a single-copy *CEN* plasmid expressing an mCherry (mCh)-tagged reporter, like Edc3 for P-bodies or Pbp1 for stress granules. These strains were then grown in an SC-based minimal medium at 30° and examined on the indicated days. Where indicated, these mCh-tagged reporters were integrated into the genome of the appropriate GFP-tagged strains. The strains used in this study are listed in S1 Table.

### Plasmid construction

For the *EDC3-mCh* integrating plasmid, pPHY4085, a fragment containing the second half of the *EDC3* gene and the mCh reporter were cloned into the pRS406 vector between the *Sal I* and *Xba I* restriction sites. Integration was then directed towards the *EDC3* locus by digestion with *Bam HI*. For the construction of the *PBP1-mCh* integrating plasmid, pPHY3782, a fragment encoding the Pbp1-mCh fusion protein was cloned between the *Kpn I* and *Sac I* sites in pRS406. The fragment also contained the *PBP1* promoter and the transcription terminator from the *ADH1* gene. This plasmid was then digested with *Sna BI* to direct integration to the *PBP1* locus. The plasmids used in this study are listed in S2 Table.

### Fluorescence microscopy

Cells expressing the appropriate fusion construct(s) were grown as indicated and then collected by centrifugation, re-suspended in the appropriate medium, and spotted onto microscope slides as described [31, 44, 45]. The cells were then imaged with a 100x/1.45 numerical aperture Plan-Apo objective lens on a Nikon Eclipse Ti inverted microscope (Nikon, Melville, NY) equipped with an Andor Zyla digital camera and the appropriate Nikon HC filter sets. The data shown indicate the fraction of cells that contain a bright fluorescent focus. In general, the cells had one or two P-body or stress granule foci per cell, respectively. For the quantitation, the data in all cases represent the average of two or more experiments where at least one hundred cells were examined in each replicate. The error bars in the graphs indicate the standard deviations for the data shown. The percentage of colocalization for any two reporters was calculated by scoring the proportion of coincident foci in those cells that possessed at least one of each type of focus [18, 40]. The final merged images were created with the Image J software package. All images within a particular figure panel were processed in precisely the same manner and thus the relative fluorescent intensity of foci and physical attributes of the cells (including size) can be directly compared. The reporters used in this study included the following proteins that have been localized to either P-bodies or stress granules. For P-bodies, we examined Dcp2, the catalytic subunit of the primary decapping enzyme in eukaryotes; Pat1, a key scaffolding protein that has also been found to act as a decapping enhancer; and the decapping enhancer, Edc3 [14, 46–48]. For stress granules, we examined the polyA-binding protein, Pab1; the Pab1-binding protein, Pbp1; and two additional reporters, Pbp4 and Lsm12 [37, 38, 49].

## Results

### Stress granule induction occurs in response to a decrease in osmotic support

**Fig 1:**
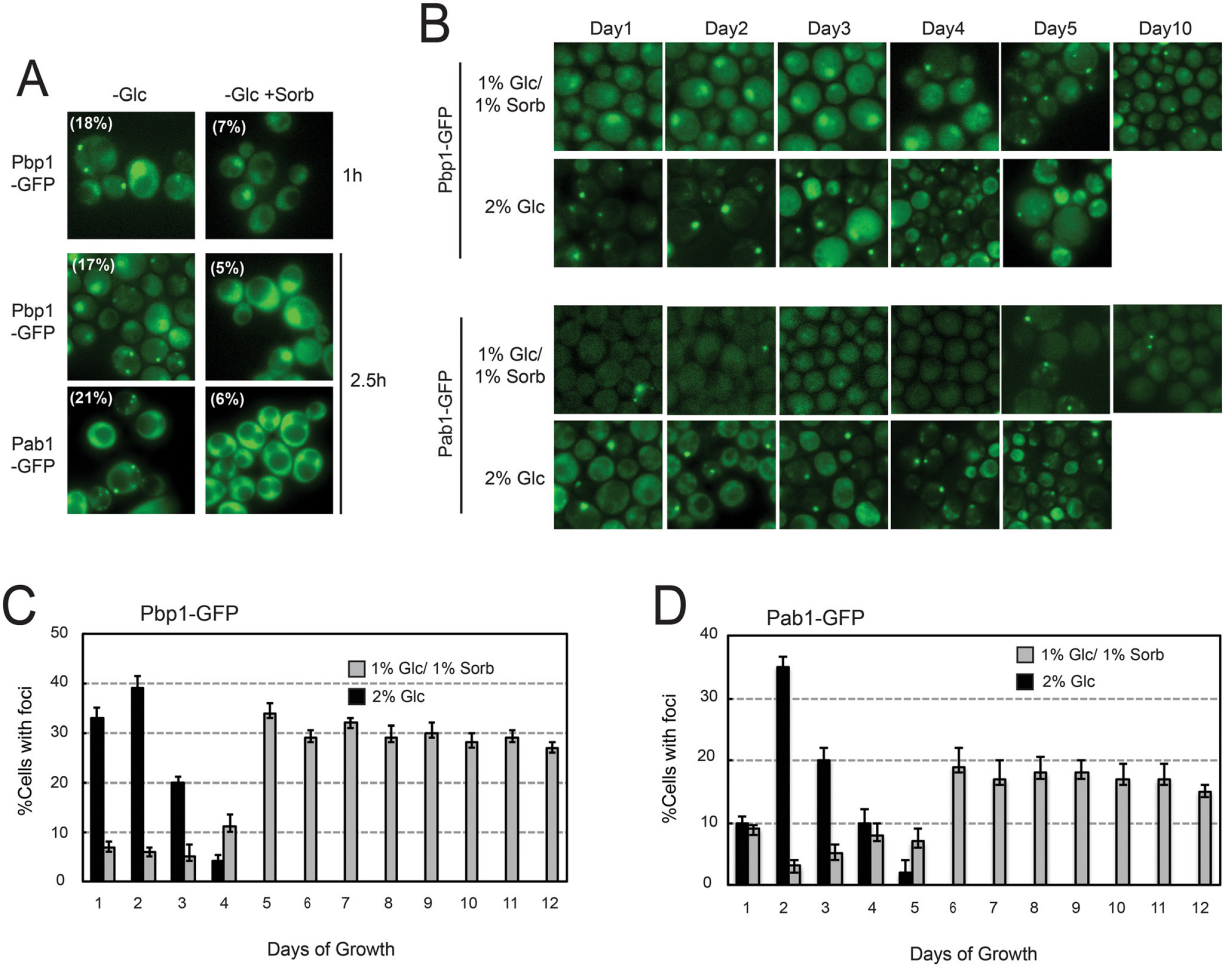
Stress granule formation in glucose-limiting conditions was suppressed by the presence of sorbitol. (A) The addition of sorbitol suppresses the stress granule induction observed upon an acute glucose starvation. Log phase wild-type cells expressing the indicated GFP-tagged stress granule reporters were transferred from SC minimal medium containing 2% glucose to the same medium lacking this sugar. After the indicated time, the cells were visualized by fluorescence microscopy. (B-D) Wild-type cells were examined by fluorescence microscopy after the indicated days of growth in SC-glucose minimal medium. The cells expressed GFP-tagged versions of either Pbp1 or Pab1. The quantitation of the microscopy data is presented in panels C and D. Note that the larger structures exhibiting more diffuse Pbp1-GFP fluorescence correspond to the nucleus (see S1B Fig) [18].

**Fig 2:**
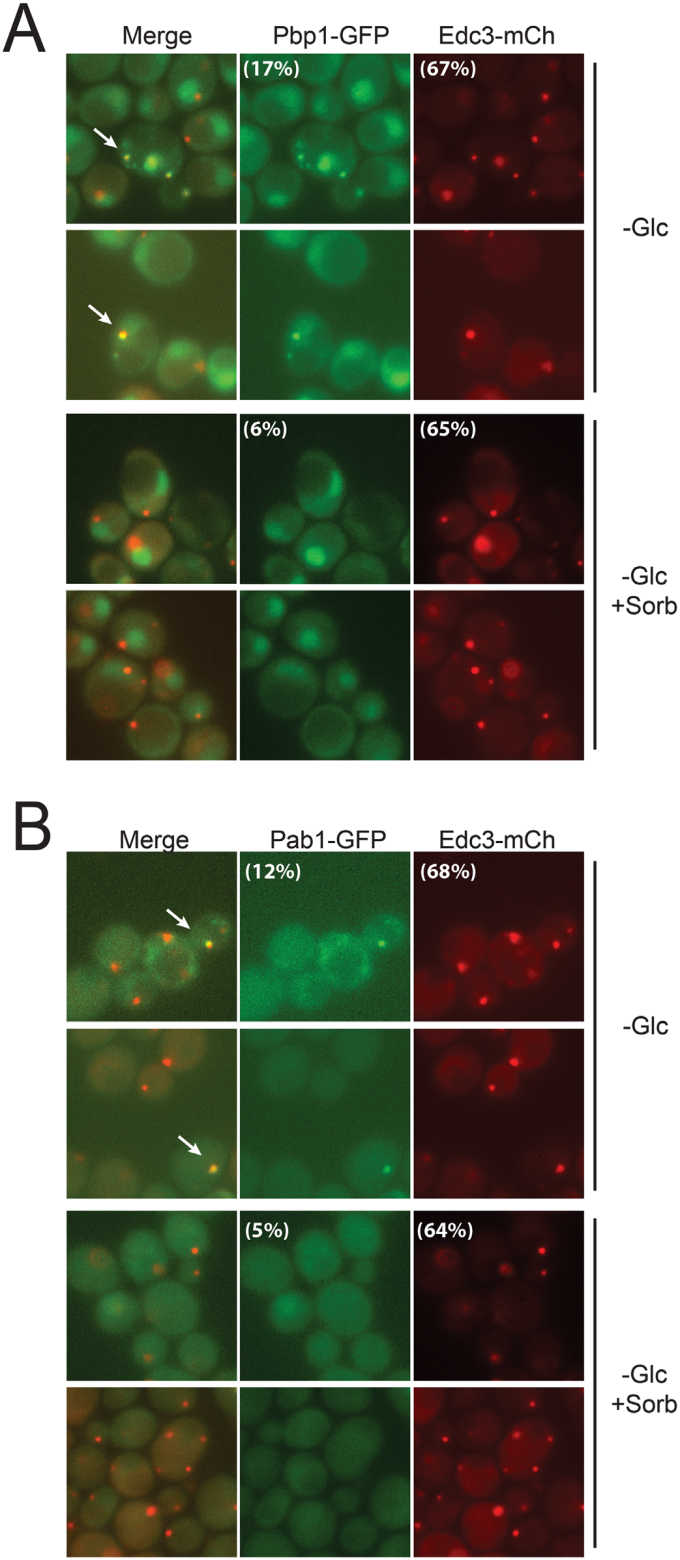
Stress granule and P-body proteins localized to the same cytoplasmic foci after transfer to a minimal medium lacking glucose. Log phase cells expressing the indicated reporter proteins were transferred to SC minimal medium lacking glucose for 2.5 hrs. The cells were then visualized by fluorescence microscopy. Pbp1 (A) and Pab1 (B) are reporters for stress granules and Edc3 for P-bodies. Examples of the merged granules are indicated by the white arrows. Note that the presence of sorbitol suppressed stress granule formation but had no significant effect on P-body induction. The fraction of cells containing a stress granule or P-body focus is indicated by the numbers in parentheses. Two fields of cells are shown for each condition examined.

One of the most common ways to induce stress granules in *S*. *cerevisiae* is to transfer cells from a growth medium with glucose to the same medium lacking this sugar (Fig 1A). These experiments have led to a model proposing that stress granule assembly is triggered by an acute starvation for glucose. However this induction is efficient only in minimal media like the traditional SC growth medium. Very few stress granules are observed when log phase cells growing in the rich medium, YPD, are transferred to YP lacking glucose (S1 Fig). This latter result suggested that the observed induction might be due to other variables in the experiment. In particular, we noted that this regimen also involved a change in the level of osmotic support. In essence, the cells were being transferred to a hypoosomotic solution and we reasoned that this change could be the trigger for stress granule formation. The more complex makeup of the rich medium, YP, may provide enough osmotic support to prevent granule formation. To test this possibility, we added 2% sorbitol to the SC-based glucose starvation medium and repeated the induction experiments. Sorbitol is a hexose sugar that is poorly metabolized by *S*. *cerevisiae* and has been used to provide osmotic support in a variety of experimental contexts [50–52]. We found that the presence of sorbitol resulted in a significant decrease in stress granule induction with the fraction of cells containing foci typically decreasing by approximately three-fold (Fig 1A). This suppression was specific to stress granules as the presence of sorbitol had no effect on the appearance of a second RNP granule, the P-body (Fig 2). These data therefore suggested that a decrease in osmotic support was an important contributing factor to the stress granule induction observed with this experimental regimen. The foci that remain when sorbitol is present could result from a failure to completely suppress the osmotic effects or a low level of induction brought on by the glucose deprivation.

### The presence of sorbitol also suppresses the stress granule induction that occurs following the diauxic shift in minimal medium

**Fig 3:**
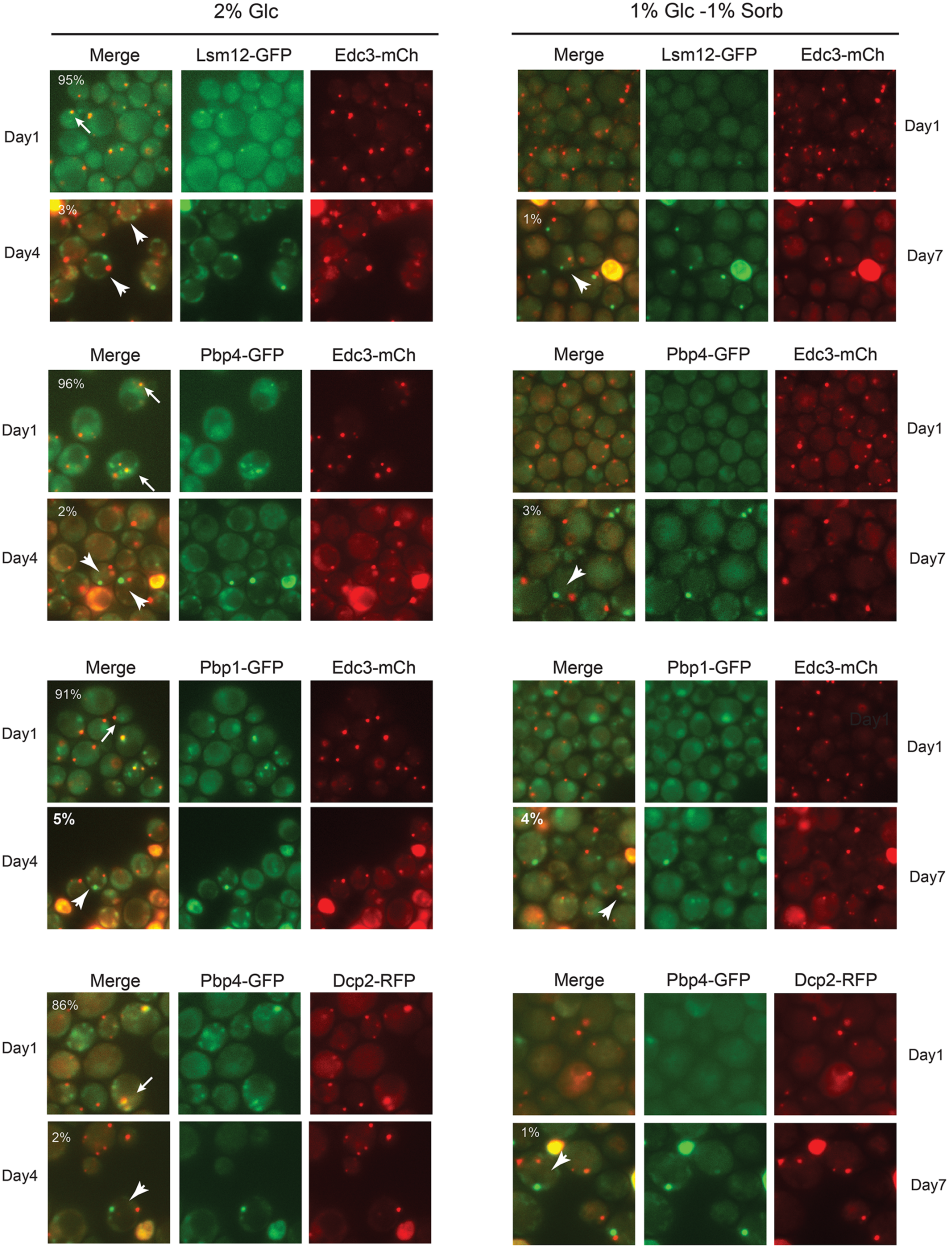
Hybrid-bodies formed transiently in cells that had just passed through the diauxic shift. Wild-type cells expressing the indicated pairs of stress granule and P-body reporters were grown in SC minimal media containing either 2% glucose or a mixture of 1% glucose and 1% sorbitol. The cells were examined by fluorescence microscopy after either 1, 4 or 7 days of growth in these respective media. The stress granule reporters, Lsm12, Pbp4 and Pbp1, were tagged with GFP and the markers for the P-body, Edc3 and Dcp2, with mCherry. The numbers indicated in the top left corner of the merged image panels indicate the relative level of colocalization observed for the two reporters present. The white arrows point out cells with colocalized reporters (containing hybrid-bodies) whereas the white arrowheads indicate cells with separate P-body and stress granule foci.

Our previous work showed that stress granules are not induced in standard YPD cultures until cells begin to enter into stationary phase [18]. This transition occurs after approximately 5 to 6 days of growth in this rich medium [53, 54]. In contrast, we found here that stress granules were induced after 1 to 2 days of growth in SC-glucose minimal medium (Fig 1B). At this time, the glucose is largely depleted from the medium and cells have progressed through a metabolic transition known as the diauxic shift. During this transition, cells switch from the fermentation of the available glucose to respiration of the ethanol produced during this period of fermentative growth [53, 55]. Since the presence of stress granules here was again correlated with the depletion of glucose, we tested whether this induction was also suppressed by the addition of sorbitol. For these experiments, a growth medium containing a mixture of 1% glucose and 1% sorbitol was used instead of the standard 2% glucose. We found that the presence of sorbitol had an even stronger suppressive effect on stress granule formation in this context; in general, we observed 5 to 10-fold fewer foci in this supplemented minimal medium (Fig 1B–1D). These suppressive effects were due to the presence of sorbitol and not the lower concentration of glucose as similar numbers of stress granules were observed in cells grown in media containing only 1% glucose (S2 Fig). Stress granules did begin to appear in more significant numbers after 4 to 5 additional days of growth in the sorbitol-supplemented medium, a time commensurate with the entry into stationary phase (Fig 1B–1D). Thus, the cells in this sorbitol-containing medium resembled those growing in the rich medium, YPD (S3A Fig) [18]. This suppression was again specific to stress granules as there were no significant effects on the induction of P-bodies (Fig 3). P-bodies normally appear after 1d of growth in both minimal and rich media in response to the declining levels of glucose [18, 33]. Therefore, although the decrease in osmotic support was occurring at a more gradual pace, this change may again be responsible for the observed induction of stress granule foci.

### Stress granule and P-body proteins are found in the same hybrid body in response to a decrease in osmotic support

Although previous work has suggested a number of potential interactions between P-bodies and stress granules [7, 32], these RNP structures are generally considered to be distinct compartments in the cytoplasm. Consistent with this, we previously observed low levels of colocalization between P-body and stress granule reporters in stationary phase cells [18, 31]. Similar values have been reported elsewhere for a variety of induction conditions although there have been studies where more significant colocalization was found [56–58]. In addition, other studies have found that certain proteins localize to both of these RNP structures to varying degrees [31, 49, 58]. However, in contrast with this previous work, we found that core stress granule and P-body constituents exhibited very high levels of colocalization with both of the experimental regimens described here. For example, the levels of coincidence for Pbp1 and Edc3 generally approached 90% for the stress granules that appeared after the diauxic shift (Fig 3). The extent of colocalization was more varied for the acute glucose starvation regimen but was typically between 70 to 95%. However, stress granule induction in general is much more variable with this latter protocol (see Discussion). For the particular experiments shown in Fig 2, we observed 85–95% colocalization between Edc3 and either Pbp1 or Pab1. A high degree of colocalization was seen with all reporter pairs examined indicating that this phenomenon was not limited to particular granule constituents (Fig 3). This colocalization was apparently specific to these two RNP granules as components of other cytoplasmic foci were not associated with this hybrid-body. The other structures examined included actin bodies and the novel protein kinase foci recently identified in yeast cells [18, 59]. Altogether, these data are most consistent with hypoosmotic stress inducing a type of merged or hybrid granule that contains the constituents of both P-bodies and stress granules.

**Fig 4:**
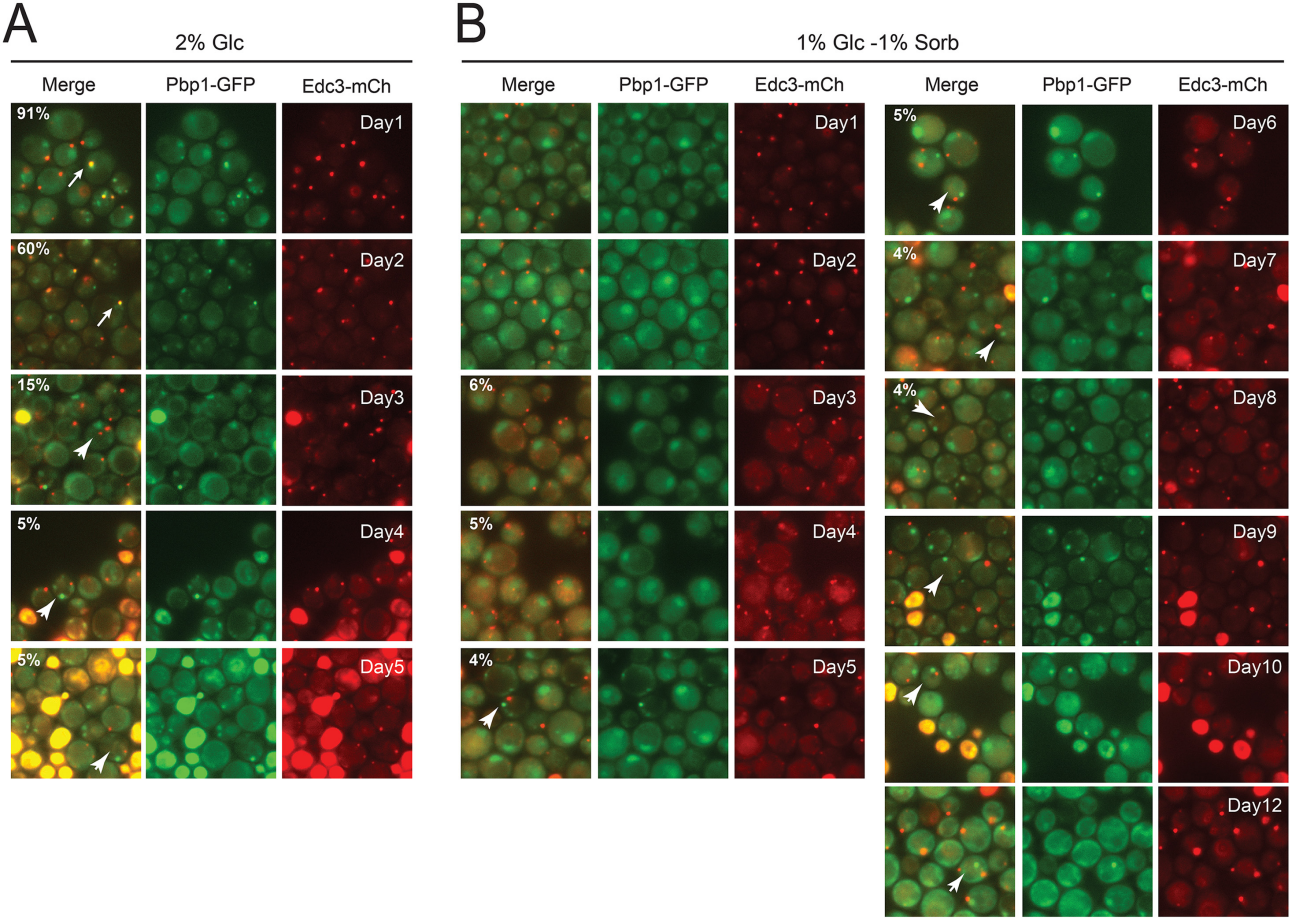
Hybrid-bodies were present only transiently in cells. Wild-type cells were grown in SC minimal media containing either 2% glucose or a mixture of 1% glucose and 1% sorbitol for the indicated number of days before being examined by fluorescence microscopy. The numbers in the top left corner of the merged image panels indicate the relative level of colocalization observed for the stress granule (Pbp1-GFP) and P-body (Edc3-mCh) reporters present. The white arrows point out cells with colocalized reporters (containing hybrid-bodies) whereas the white arrowheads indicate cells with separate P-body and stress granule foci. The cells grown in the medium containing 2% glucose exhibited a significant level of cell death with increasing time and thus were examined only during the first five days of culture growth.

Interestingly, these hybrid-bodies appear to be transient structures as P-body and stress granule colocalization was found to decrease steadily from a maximum at day 1 to minima after 4–5 days of growth (Figs 3 and 4). The colocalization at these later times was typically less than 5%, an indication that these two granules were largely distinct structures at this time. These data are therefore consistent with previous studies that examined these RNP granules in stationary phase cells [18, 56]. A more detailed timeline for Edc3 (P-bodies) and Pbp1 (stress granules) localization is shown in Fig 4. Although the underlying mechanisms responsible for the ultimate resolution of these two granules are not yet clear, it is likely that the dynamic nature of these structures contributes to this process. In particular, previous data have suggested that proteins (and mRNAs) are constantly exchanging between the cytoplasm and these RNP granules [6, 7, 19]. Over time, the cells presumably adapt to the osmotic stress and granule proteins that were previously in the merged structure would now assemble into distinct P-body and stress granule foci.

Finally, the data here indicated that the presence of stress granule proteins in the hybrid-bodies was not dependent upon prior P-body formation. Specifically, we found that foci containing stress granule reporters were detected at similar levels in a mutant, *pat1*Δ, that is defective for P-body assembly [18]. For example, Pbp1 foci were present in 33 and 35% of wild-type and *pat1*Δ cells, respectively, after 1d of growth in minimal medium (S3B Fig). A similar result was reported previously for an acute glucose starvation and this was confirmed here [18]. These results therefore suggest that P-body and stress granule proteins are independently recruited to a common cytoplasmic granule in response to a hypoosmotic stress.

## Discussion

This study examined the conditions that induce SG and P-body foci in *S*. *cerevisiae* and the relationship that exists between these two conserved RNP granules. The data here make three important contributions to our understanding of P-body and stress granule biology. First, they indicate that stress granule formation in yeast can be triggered by an exposure to a hypoosmotic stress. In these studies, cells were transferred from a minimal medium containing glucose to the same medium lacking this sugar. These results were previously interpreted as evidence for glucose deprivation being the primary trigger for granule formation. However, the results here suggest that it is the change in osmotic conditions that is responsible for stress granule induction. This observation may also provide an explanation for the considerable variability observed with this experimental regimen and the fact that several research groups have been unable to observe significant stress granule formation upon glucose starvation. Since the induction appears to occur in response to changes in solution osmolarity, differences in medium preparation could be responsible for the varying levels of granule formation.

The second finding is that P-body and stress granule proteins localize to the same cytoplasmic foci and form a novel hybrid-body in response to this particular stress condition. The existence of this hybrid-body offers a potential explanation for conflicting reports that have placed proteins like Pab1 alternatively in P-bodies and stress granules [38, 49, 57]. Finally, the data indicate that these hybrid-bodies are transient structures that exist for only a limited period of time. As the cells presumably adapt to the stress, the P-body and stress granule proteins would relocate to their respective distinct structures in the cytoplasm. This relocalization is very likely to depend upon the active shuttling that has been observed between these RNP structures and the respective cytoplasmic pools of granule constituents.

The observation that stress granules are induced by a hypoosmotic stress, and not glucose deprivation, offers a potential explanation for previous data indicating that stress granule formation is not affected by changes in PKA signaling activity [18]. The PKA pathway in *S*. *cerevisiae* is regulated by glucose availability and generally has a role in glucose-mediated responses [60, 61]. For example, constitutive signaling through this pathway has been shown to inhibit the P-body formation that occurs in response to glucose deprivation [18, 40]. These results however beg the question as to what signaling pathways might be involved in the response to the hypoosmotic stress described here. Unfortunately, very little is presently known about the yeast cell response to such hypotonic stress conditions. In contrast, there is a significant literature describing the role of the Hog1 MAP kinase in the response to a hyperosmotic stress [62, 63]. In particular, the activation of this enzyme results in the increased cytoplasmic production of glycerol that effectively counteracts the hyperosmotic stress [63]. There is currently no known role for Hog1 in the response to a hypotonic stress and we found that stress granule induction was normal in a *hog1*Δ mutant after one day of growth in minimal medium (data not shown). Thus, additional work is needed to identify the signaling pathway controlling stress granule induction under these conditions.

It is important to note that although both P-body and stress granule proteins were found in the hybrid-body that the underlying reasons for this localization appear to be distinct. In particular, our data indicate that recruitment of P-body proteins was induced by a decrease in glucose concentration and not the change in osmotic conditions. Interestingly, previous work has shown that P-bodies can be induced by changes in medium osmolarity but the conditions used in this prior study were different from those described here [33]. A key question that remains is whether the formation of these hybrid-bodies (or stress granules alone) is important for cell survival during this period of stress. Perhaps the best way to test this possibility would be with a mutant specifically defective for the formation of hybrid-bodies. Unfortunately, such a mutant is not available at this time. However, our previous work has identified a mutant, *ubp3*Δ, that is defective for stress granule assembly during the entry into stationary phase [64, 65]. *UBP3* encodes a deubiquitinating enzyme that removes ubiquitin moieties from a specific set of modified proteins [66]. The *ubp3*Δ mutant forms fewer stress granules than the wild-type control after one day of growth in minimal medium [65]. Although these mutants exhibited a diminished long-term survival in stationary phase, there were no significant viability defects after one to two days of growth in this minimal medium. This would correspond to the time when hybrid bodies would be expected to be present in the mutant cells. A determination of the importance of the hybrid-body for survival during an acute exposure to a hypotonic stress will require the identification of a mutant that is specifically defective for granule formation at this time.

Finally, it is important that we place the observations here into the larger context of the different nonmembranous compartments that coexist within the eukaryotic cell. In particular, our data demonstrate that the composition, and even identity, of an RNP granule can vary significantly with different induction conditions. This is an important observation as the physiological roles of these structures are likely determined by the protein complement present within each granule. An altered protein constituency would be expected to result in a different set of activities for that RNP structure. As a result, we need to determine why P-body and stress granule proteins localize to the same foci under the conditions described here and how the functions of the individual granules might differ in this merged configuration. In addition, it is not clear whether this convergence is unique to hypoosmotic stress or whether it is a more general phenomenon that will be observed with these and perhaps other cytoplasmic granules. Answering these and related questions is likely to provide novel insight into the biological roles these RNP structures play in the eukaryotic cell.

## Supporting Information

S1 Fig Stress granules were not induced in the rich medium, YPAD. (A) Cells expressing the indicated reporters were transferred from YPA medium containing 2% glucose to the same medium lacking this sugar. The cells were then visualized by fluorescence microscopy. (B) Pbp1 was localized to the nuclear compartment of yeast cells. Cells expressing Pbp1-GFP and the nuclear reporter, histone H2B-mCh, were examined by fluorescence microscopy.
(TIF)

S2 Fig Hybrid-bodies were induced after one day of growth in SC minimal medium containing 1% glucose. Wild-type cells expressing the Pat1-GFP (P-body) and Pbp1-mCh (stress granule) reporters were grown in SC minimal media containing either 1% or 2% glucose for the indicated number of days before being examined by fluorescence microscopy. The numbers in the top left corner of the merged image panels indicate the relative level of colocalization observed for the two reporters.
(TIF)

S3 Fig Lower levels of P-body and stress granule colocalization were observed in the rich medium, YPAD. (A) Wild-type cells expressing the Edc3-GFP (P-body) and Pbp1-mCh (stress granule) reporters were grown in YPAD medium for the indicated number of days before being examined by fluorescence microscopy. The numbers in the top left corner of the merged image panels indicate the relative level of colocalization observed for the two reporters. (B) Stress granule formation occurred at the same rate in *pat1Δ* cells that are defective for P-body assembly. Wild-type and *pat1Δ* cells expressing the Pbp1-GFP reporter were grown for 1 day in SC minimal medium containing 2% glucose before being examined by fluorescence microscopy. The numbers in the top left corner indicate the fraction of cells containing a Pbp1-GFP focus.
(TIF)

S1 Table Plasmids used in this study. (DOCX)

S2 Table Yeast strains used in this study. (DOCX)
