# Supplementary figures and images for: A Hybrid-Body Containing Constituents of Both P-Bodies and Stress Granules Forms in Response to Hypoosmotic Stress in Saccharomyces cerevisiae
Source: PLoS One. 2016 Jun 30;11(6):e0158776. doi: 10.1371/journal.pone.0158776 (PMC4928847; doi:10.1371/journal.pone.0158776)

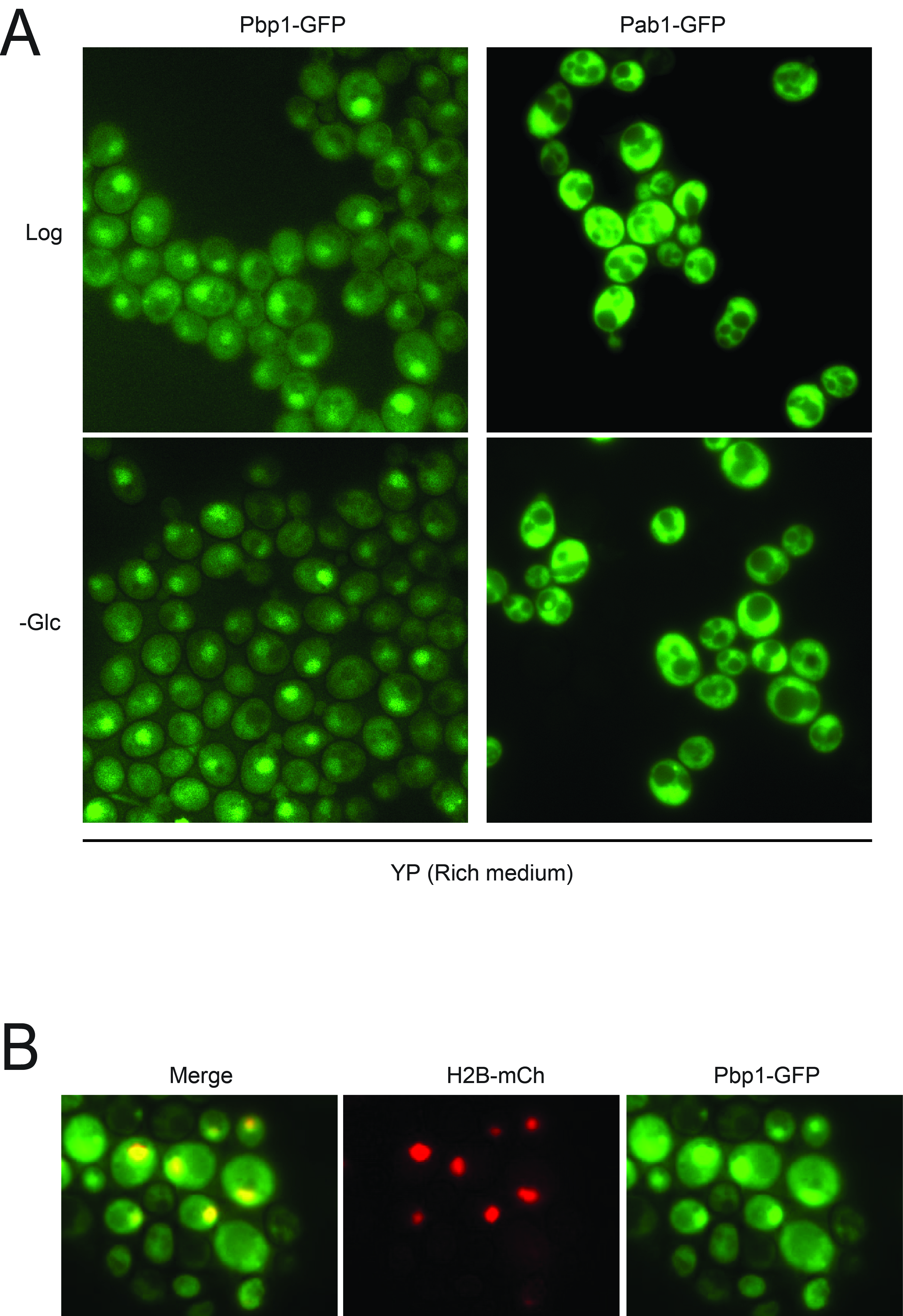

Supplement: S1 Fig — (A) Cells expressing the indicated reporters were transferred from YPA medium containing 2% glucose to the same medium lacking this sugar. The cells were then visualized by fluorescence microscopy. (B) Pbp1 was localized to the nuclear compartment of yeast cells. Cells expressing Pbp1-GFP and the nuclear reporter, histone H2B-mCh, were examined by fluorescence microscopy. (TIF) [file pone.0158776.s001.tif]

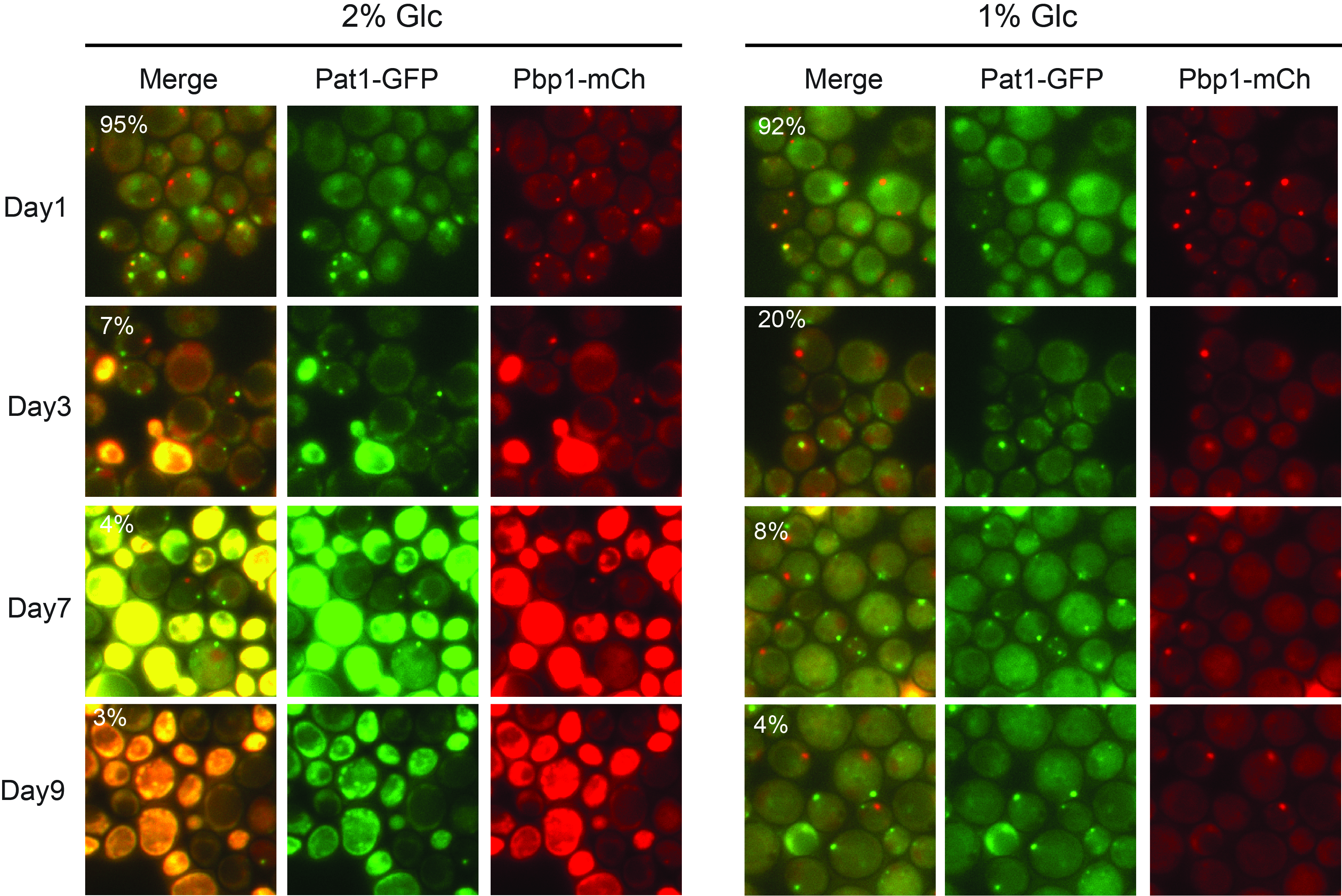

Supplement: S2 Fig — Wild-type cells expressing the Pat1-GFP (P-body) and Pbp1-mCh (stress granule) reporters were grown in SC minimal media containing either 1% or 2% glucose for the indicated number of days before being examined by fluorescence microscopy. The numbers in the top left corner of the merged image panels indicate the relative level of colocalization observed for the two reporters. (TIF) [file pone.0158776.s002.tif]

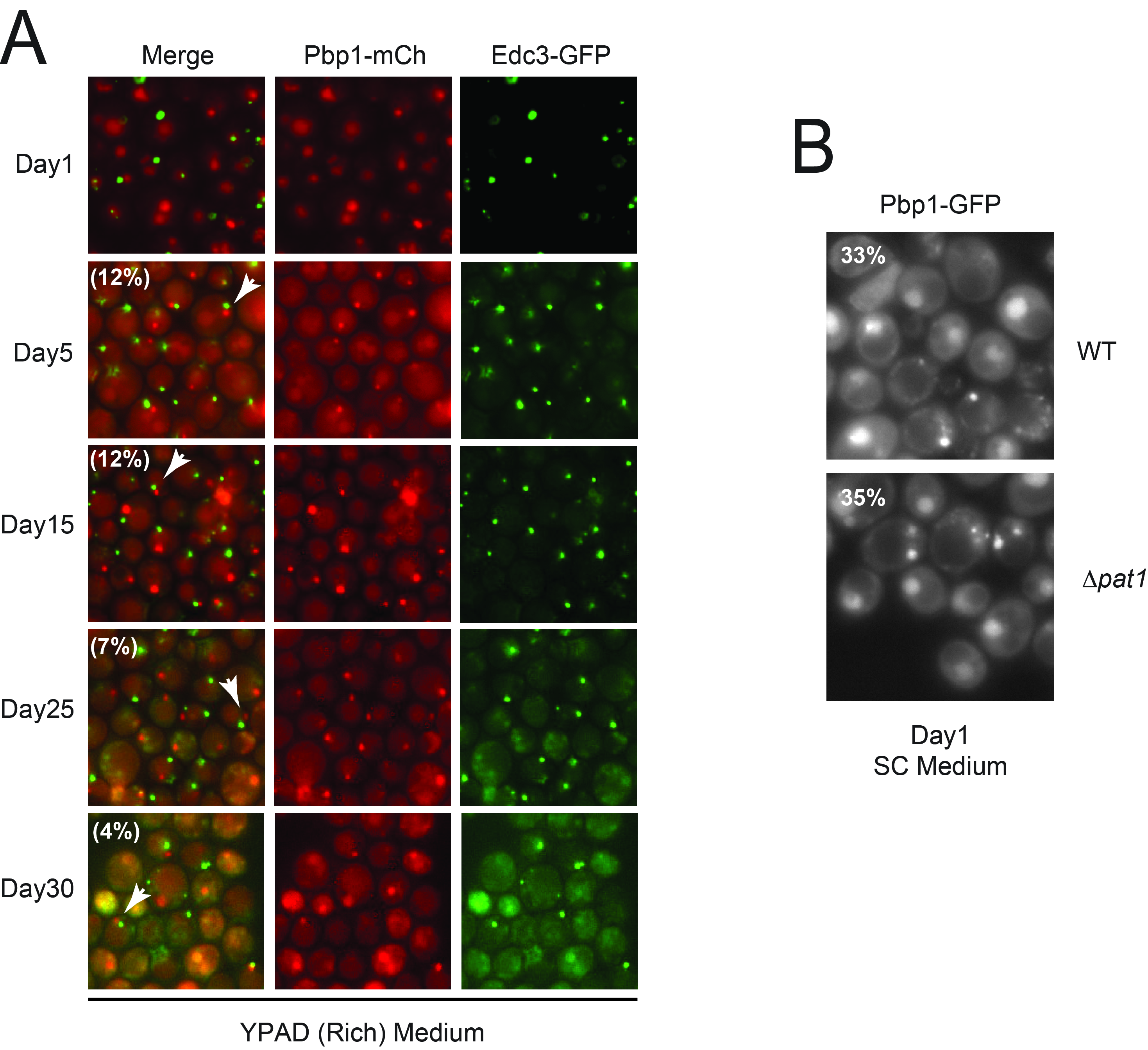

Supplement: S3 Fig — (A) Wild-type cells expressing the Edc3-GFP (P-body) and Pbp1-mCh (stress granule) reporters were grown in YPAD medium for the indicated number of days before being examined by fluorescence microscopy. The numbers in the top left corner of the merged image panels indicate the relative level of colocalization observed for the two reporters. (B) Stress granule formation occurred at the same rate in pat1Δ cells that are defective for P-body assembly. Wild-type and pat1Δ cells expressing the Pbp1-GFP reporter were grown for 1 day in SC minimal medium containing 2% glucose before being examined by fluorescence microscopy. The numbers in the top left corner indicate the fraction of cells containing a Pbp1-GFP focus. (TIF) [file pone.0158776.s003.tif]
