# Supplementary material for: A Hybrid-Body Containing Constituents of Both P-Bodies and Stress Granules Forms in Response to Hypoosmotic Stress in Saccharomyces cerevisiae
Source: PLoS One. 2016 Jun 30;11(6):e0158776. doi: 10.1371/journal.pone.0158776 (PMC4928847; doi:10.1371/journal.pone.0158776)
Supplement: S1 Table — (DOCX) [file pone.0158776.s004.docx]

**Table S1. Plasmids used in this study.**

| **PHY Name** | **Original Name** | **Relevant Details** | **Source** |
| --- | --- | --- | --- |
| pPHY3660 | pRP1574 | *EDC3-mCherry* | Dr. Roy Parker |
| pPHY3702 | pOE79 | *ADH2pro-H2B-mCherry* | Dr. James Hopper |
| pPHY3714 |  | *DCP2-RFP-PGK1_term_*  (in pPRS415; *CEN, LEU2*) | Dr. C. De Virgillio |
| pPHY3782 |  | *PBP1-mCherry-ADH2_term_* (in pRS416; *CEN, URA3*) | Lab collection |
| pPHY3830 |  | *PBP1-mCherry-ADH2_term_* (in pRS406; *CEN, URA3*) | This study |
| pPHY4085 |  | *EDC3-mCherry-ADH2_term_* (in pRS406; *URA3*) | Dr. Regina Nostramo |
