# Supplementary material for: A Hybrid-Body Containing Constituents of Both P-Bodies and Stress Granules Forms in Response to Hypoosmotic Stress in Saccharomyces cerevisiae
Source: PLoS One. 2016 Jun 30;11(6):e0158776. doi: 10.1371/journal.pone.0158776 (PMC4928847; doi:10.1371/journal.pone.0158776)
Supplement: S2 Table — (DOCX) [file pone.0158776.s005.docx]

**Table S2. Yeast strains used in this study**

| BY4741 (PHY4231) | *MATa hisΔ1 leu2Δ0 met15Δ0 ura3Δ0* | Open Biosystems |
| --- | --- | --- |
| PHY4773 | *MATa his3Δ1 leu2Δ0 met15Δ0 ura3Δ0* *DCP2-GFP* | Invitrogen |
| PHY4899 | *MATa his3Δ1 leu2Δ0 met15Δ0 ura3Δ0* *PAB1-GFP* | Invitrogen |
| PHY4900 | *MATa* *his3Δ1 leu2Δ0 met15Δ0 ura3Δ0* *PBP1-GFP* | Invitrogen |
| PHY4939 | *MATa* *his3Δ1 leu2Δ0 met15Δ0 ura3Δ0* *PBP4-GFP* | Invitrogen |
| PHY4940 | *MATa his3Δ1 leu2Δ0 met15Δ0 ura3Δ0* *PUB1-GFP* | Invitrogen |
| PHY4941 | *MATa his3Δ1 leu2Δ0 met15Δ0 ura3Δ0* *LSM12-GF*P | Invitrogen |
| PHY4942 | *MATa his3Δ1 leu2Δ0 met15Δ0 ura3Δ0* *PAT1-GFP* | Invitrogen |
| PHY6008 | PHY4942 *PBP1-mCh::URA3* | This study |
| PHY6012 | PHY4773 *PBP1-mCh::URA3* | This study |
| PHY5475 | BY4741 *EDC3-GFP::HIS3* | This study |
| PHY5492 | PHY5475 *PBP1-mCh::URA3* | This study |
| PHY6445 | PHY4900 *EDC3-mCh::URA3* | This study |
